# Multiplex Molecular Stool Testing Rarely Impacts Antimicrobial Treatment Decisions More Than Three Days After Admission

**DOI:** 10.7759/cureus.14784

**Published:** 2021-04-30

**Authors:** Abdul Aleem, Gabriela Firak, Amy K Slenker

**Affiliations:** 1 Gastroenterology and Hepatology, Lehigh Valley Health Network, Allentown, USA; 2 Internal Medicine, Tower Health-Reading Hospital, Reading, USA; 3 Infectious Diseases, Lehigh Valley Health Network, Allentown, USA

**Keywords:** antimicrobial treatment decisions, molecular stool testing, c. difficile, diarrhea, 3-day rule

## Abstract

Background

Acute diarrheal illness in the United States is a significant cause of healthcare utilization and hospitalizations. For patients who develop diarrhea while hospitalized, testing for pathogens other than *Clostridium difficile* (*C. difficile*) using conventional stool testing is low yield. Newer testing modalities for infectious diarrhea such as the* *multiplex molecular stool testing provide an improved detection rate and a faster turn-around time compared to conventional stool testing.

Methods

We retrospectively examined the use of a multiplex molecular stool test at our institution for all hospital encounters over a two-year period to determine which organisms were identified ≤ 3 days and > 3 days after admission.

Results

A total of 2032 patients underwent multiplex molecular stool testing during the study period, with 1698 (83.6%) performed ≤ 3 days and 334 (16.3%) > 3 days after admission. An enteric non-*C. difficile* pathogen was identified more frequently when patients were tested ≤ 3 days after admission (350, 20.6%) as compared to > 3 days after admission (38, 11.4%, *p*<0.0001). Excluding coinfections, *C. difficile* was identified more frequently when patients were tested > 3 days after admission (64, 20.3%) versus another organism (30, 9.0%) (*p*<0.0001). Of those patients with a non-*C. difficile* pathogen identified > 3 days after admission, a bacterial pathogen amenable to treatment was only identified in 6% (21) of patients.

Conclusion

Multiplex molecular stool testing for patients tested > 3 days after admission is a low yield of information that could guide antimicrobial treatment decisions, and *C. difficile* testing is more useful in this clinical situation.

## Introduction

Acute diarrheal illness is a significant cause of morbidity and mortality worldwide, with the most common infectious etiologies being *Norovirus*, *Campylobacter spp*., and non-typhoidal *Salmonella *infections [1]. In resource-rich countries, such as the United States, diarrhea illness is a rare cause of death but is a significant source of outpatient care utilization and hospitalizations [2]. In the US, diagnostic testing for community-onset diarrhea should be performed in patients with fever, bloody or mucoid stools, severe symptoms, or an immunocompromised state who would benefit from antimicrobial therapy [3]. 

Conversely, for patients who develop diarrhea while hospitalized, testing for infectious etiologies via stool culture for enteric pathogens is low value, as well as costly [4, 5]. Prior studies with conventional stool testing methods have shown that in patients who develop diarrhea while hospitalized, testing for pathogens other than *Clostridium difficile* (*C. difficile*) is low yield when performed > 3 days after admission [6], which has led to the adoption of the “3-day rule” by many laboratories. 

Conventional testing methods are increasingly being replaced by multiplex molecular stool testing, which provides an improved detection rate for multiple pathogens simultaneously with a high sensitivity and a rapid turnaround time [7-9]. However, it is not clear if the “3-day rule” should be applied to this new test. Prior studies on this topic revealed the percent positivity rates by molecular testing for stool samples collected 72 hours after admission ranged from 6% to 14.6% [10-11].

Our institution began using multiplex molecular stool testing in place of routine stool culture for the diagnosis of suspected infectious diarrhea on January 5, 2015. This test identifies 22 different bacterial, viral, and parasitic stool pathogens, including *C. difficile*. In this study, we retrospectively examined the use of a multiplex molecular stool test for all hospital encounters over a two-year period to determine which organisms were identified in patients who are tested after being hospitalized ≤ 3 days and > 3 days. We also aimed to evaluate if prior guidance regarding a “3-day rule” is applicable in the molecular testing age.

## Materials and methods

This was a retrospective chart review study of all adult patients 18 years or older who presented to Lehigh Valley Health Network (LVHN) Cedar Crest or Muhlenberg campuses from January 5, 2015, to January 4, 2017, who had a multiplex molecular stool test performed during their hospital encounter. The patient’s charts were reviewed to obtain age, gender, admission date, stool testing date, and the result of stool testing. Repeat testing during the same hospital admission was not included. Patients with indeterminate results were excluded. 

The specimens were received by the laboratory in Cary-Blair enteric transport medium and were tested via the FilmArray Gastrointestinal (GI) Panel (BioFire Inc., Salt Lake City, UT, USA) according to the package insert. The FilmArray® GI Panel looks for the following pathogens: *Campylobacter *(*jejuni*, *coli*, and *upsaliensis*), *Clostridium difficile *(Toxin A/B), *Plesiomonas shigelloides*, *Salmonella*, *Vibrio *(*parahaemolyticus*, *vulnificus*, and *cholerae*), *Yersinia enterocolitica*, Enteroaggregative *Escherichia coli* (EAEC), Enteropathogenic *Escherichia coli *(EPEC), Enterotoxigenic *Escherichia coli *(ETEC), Shiga-like toxin-producing *Escherichia coli* (STEC), *Escherichia coli* O157, Shigella/Enteroinvasive *Escherichia coli* (EIEC), *Cryptosporidium*, *Cyclospora cayetanensis*, *Entamoeba histolytica*, *Giardia lamblia*, *Adenovirus *F 40/41, *Astrovirus*, *Norovirus *GI/GII, *Rotavirus *A, and *Sapovirus *(I, II, IV, and V). 

The primary endpoint was to determine if there is a significant difference in the percentage of tests resulting with enteric (non-*C. difficile*) pathogens if the testing was performed ≤ 3 or > 3 days after hospital admission. A secondary endpoint included determining if there was a difference in the percentage of tests resulting with *C. difficile *versus other enteric, non-*C. difficile *pathogens if the testing was performed > 3 days after hospital admission. 

Statistical analysis

Continuous variables were described using the median and interquartile range (IQR), and categorical variables were described with frequency and percentage. When excluding coinfections, the Chi-Square test was used as patients could only be placed into one group. When including co-infections, McNemar’s test was used as patients could have tested positive for both enteric non-*C. difficile *pathogens and *C. difficile*. All statistical analyses were conducted using SAS 9.3 (SAS Institute, Cary, NC, USA).

## Results

The study sample consisted of 2032 patients, of which the median age was 64 (IQR 51-76) years, and 854 were males (42%). The median number of days into the patient’s hospital stay that the multiplex molecular stool test was performed was 1 (range 0-63) day. In total, 703 (34.6%) patients tested positive for any pathogen: 315 (15.5%) patients tested positive for *C. difficile *alone, 333 (16.4%) patients tested positive for at least one pathogen other than *C. difficile*, and 55 (2.7%) patients tested positive for both *C. difficile *and at least one other pathogen (Table 1). 

**Table 1:** Patient Demographics and Test Results Data are n(%) unless otherwise stated; percentages might not add up to 100% due to rounding. C. difficile, *Clostridium difficile*

Characteristic	Total (n=2032)	≤3 Days (n=1698)	>3 Days (n=334)
Age, years median (IQR)	64 (51-76)	63 (49-75)	67 (56-77)
Gender			
Male	854 (42.0)	699 (41.2)	155 (46.4)
Female	1178 (58.0)	999 (58.8)	179 (53.6)
Day after admission test done median (IQR)	1 (0-2)	1 (0-1)	8 (5-12)
Positive test result			
Yes	703 (34.6)	601 (35.4)	102 (30.5)
No	1329 (65.4)	1097 (64.6)	232 (69.5)
Infection status			
No infection detected	1329 (65.4)	1097 (64.6)	232 (69.5)
Infected with C. difficile only	315 (15.5)	251 (14.8)	64 (19.2)
Infected with other (non-C. difficile) pathogen only	333 (16.4)	303 (17.8)	30 (9.0)
Infected with both C. difficile and other pathogens (coinfection)	55 (2.7)	47 (2.8)	8 (2.4)

The frequency and percentage of patients who tested positive for all pathogens included in the multiplex molecular stool assay, as well as the breakdown of the organism identified as compared to the day of testing (≤ 3 days versus > 3 days after admission), is shown in Table 2. An enteric, non-*C. difficile *pathogen was identified in 350 patients (20.6%) when patients were tested ≤ 3 days after admission as compared to > 3 days after admission (38, 11.4%, *p*<.0001) (Table 2). 

**Table 2:** Positive Test Results by Pathogen and Day of Testing After Admission Data are n (%) unless otherwise stated; percentages might not add to 100% due to rounding; table includes coinfections. EAEC, enteroaggregative; EPEC, enteropathogenic; ETEC, enterotoxigenic; STEC, Shiga-like toxin-producing; EIEC, Shigella/enteroinvasive; E. Coli, *Escherichia coli*; C. difficile: *Clostridium difficile*

Pathogen	Total (n=2032)	≤3 Days (n=1698)	>3 Days (n=334)
Clostridium difficile (Toxin A/B)	370 (18.2)	298 (17.6)	72 (21.6)
Total Other Pathogen (not C. difficile)	388 (19.1)	350 (20.6)	38 (11.4)
Campylobacter (jejuni, coli, and upsaliensis)	41 (2.0)	38 (2.2)	3 (0.9)
Plesiomonas shigelloides	4 (0.2)	4 (0.2)	0
Salmonella	17 (0.8)	15 (0.9)	2 (0.6)
Vibrio (parahaemolyticus, vulnificus, and cholera)	1 (0.05)	0	1 (0.3)
Vibrio Cholera	1 (0.05)	1 (0.1)	0
E. coli EAEC	39 (1.9)	36 (2.1)	3 (0.9)
E. coli EPEC	116 (5.7)	102 (6.0)	14 (4.2)
E. coli ETEC	11 (0.5)	9 (0.5)	2 (0.6)
E. coli STEC	10 (0.5)	10 (0.6)	0
E. coli o157	10 (0.5)	8 (0.5)	2 (0.6)
E. coli EIEC	10 (0.5)	10 (0.6)	0
Cryptosporidium	10 (0.5)	10 (0.6)	0
Cyclospora cayetanesis	0	-	-
Entamoeba histolytica	0	-	-
Giardia lamblia	6 (0.3)	5 (0.3)	1 (0.3)
Adenovirus F 40/41	5 (0.3)	4 (0.2)	1 (0.3)
Astrovirus	8 (0.4)	8 (0.5)	0
Norovirus GI/GII	124 (6.1)	115 (6.8)	9 (2.7)
Rotavirus A	7 (0.3)	7 (0.4)	0
Sapovirus (I, II, IV, and V)	17 (0.8)	17 (1.0)	0
Yersinia enterocolitica	11 (0.5)	10 (0.6)	1 (0.3)

*Clostridium difficile* was the most prevalent individual organism identified in patients tested both ≤ 3 days and > 3 days after admission (298, 17.6%) versus (72, 21.6%). After excluding coinfections, patients were more likely to have *C. difficile *diagnosed if tested > 3 days as compared to ≤ 3 days after admission (64, 19.6% versus 251, 15.2%, *p*=0.0459).

After excluding coinfections, in patients who had a multiplex molecular stool test performed > 3 days after admission, *C. difficile *was identified in 64 patients (20.3%) versus other organisms (30, 9.0%) (*p*<0.0001) (Table 3). Of those, 30 patients tested > 3 days after admission, the following pathogens were identified: *Adenovirus *F40/41 (1), *Campylobacter Spp*. (2), *Escherichia coli* (EAEC) (3), *Escherichia coli* (EPEC) (10), *Escherichia coli* (ETEC) (2), *Giardia lamblia *(1), *Norovirus *GI/GII (8), *Salmonella sp*. (1), *Vibrio sp.* (1), *Yersinia enterocolitica *(1).

**Table 3:** Association Between Testing Positive for C. difficile and Testing Positive for Other Pathogens When Testing was Performed > 3 Days After Admission (Excluding Coinfections) *p*<0001; data are n (%) unless otherwise stated C. difficile, *Clostridium difficile*

	≤3 Days (n=554)	>3 Days (n=94)
Infected with C. difficile only (n=315)	251 (79.7)	64 (20.3)
Infected with another pathogen only (n=333)	303 (91.0)	30 (9.0)

## Discussion

This retrospective study reviewed the use of multiplex molecular stool testing performed ≤ 3 and > 3 days after admission at a large academic institution over a two-year period. Clinical decision support was not instituted with the transition from conventional stool testing to multiplex molecular stool testing and subsequently, it was noted that providers were using this test indiscriminately for all hospitalized patients with concern for infectious diarrhea. 

Most patients had stool testing performed early in the admission (median 1, range 0-63); however, 334 (16.3%) of patients had multiplex molecular stool testing performed > 3 days after admission. For patients tested > 3 days after admission, testing was negative in 232 (69.5%), consistent with previous findings that nosocomial diarrhea is often noninfectious in etiology [12].

Patients were more likely to be diagnosed with an enteric non-*C. difficile *pathogen if tested ≤ 3 days after admission (350, 20.6%) as compared to > 3 days after admission (38, 11.4%, *p*<.0001). This is consistent with previous data showing that stool testing for infectious pathogens is higher yield earlier in admission. Importantly, after excluding coinfections, patients who had multiplex molecular stool test performed > 3 days after admission were more likely to have *C. difficile *identified (64, 20.3%) versus another organism (30, 9.0%) (Table 1, *p*<0.0001). After a case review of those 30 patients tested > 3 days after admission who a pathogen other than *C. difficile *identified, only 21 (6%) were bacterial causes potential amenable to treatment, and of those, only 7 (2%) patients received treatment.

This strength of this study is that it is a large study reviewing the real-life application of a novel testing modality. This study provides actionable data for other institutions transitioning to multiplex molecular stool testing by supporting the implementation of clinical decision support for a “3-day rule” to discourage molecular stool testing > 3 days after admission. The limitations of this study include its retrospective nature and that it was performed at a single healthcare institution.

## Conclusions

Hospitalized patients with diarrhea tested > 3 days after admission using multiplex molecular stool testing were more likely to have *C. difficile *identified versus another enteric organism. Clinical decision support is advised to guide providers to preferentially perform *C. difficile *testing for patients hospitalized > 3 days as opposed to multiplex molecular stool testing.
